# Gut Microbiome Dysbiosis in Atopic Dermatitis: Pathogenic Mechanisms, Gut–Skin Axis Disruption, and Emerging Microbiota-Targeted Therapies

**DOI:** 10.3390/biomedicines14081711

**Published:** 2026-07-30

**Authors:** Lidia Boldeanu, Alice Elena Ghenea, Marius Bogdan Novac, Virgilios Galatis, Rodica Pădureanu, Mohamed-Zakaria Assani, Vlad Pădureanu, George G. Mitroi, Ancuța-Ramona Boicea Camen, Mihail Virgil Boldeanu

**Affiliations:** 1 Department of Microbiology, Faculty of Medicine, University of Medicine and Pharmacy of Craiova, 200349 Craiova, Romania; lidia.boldeanu@umfcv.ro (L.B.); gaman_alice@yahoo.com (A.E.G.); 2 Department of Anesthesiology and Intensive Care, University of Medicine and Pharmacy of Craiova, 200349 Craiova, Romania; marius.novac@umfcv.ro; 3 Doctoral School, University of Medicine and Pharmacy of Craiova, 200349 Craiova, Romania; mohamed.assani@umfcv.ro; 4 Department of Internal Medicine, Faculty of Medicine, University of Medicine and Pharmacy of Craiova, 200349 Craiova, Romania; rodica.padureanu@umfcv.ro (R.P.); vlad.padureanu@umfcv.ro (V.P.); 5 Department of Immunology, Faculty of Medicine, University of Medicine and Pharmacy of Craiova, 200349 Craiova, Romania; mihail.boldeanu@umfcv.ro; 6 Department of Dermatology, University of Medicine and Pharmacy of Craiova, 200349 Craiova, Romania; mitroi.george@yahoo.com; 7 Department of Occupational Medicine, University of Medicine and Pharmacy of Craiova, 200349 Craiova, Romania

**Keywords:** atopic dermatitis, gut microbiome, gut–skin axis, dysbiosis, microbial metabolites, probiotics, multi-omics

## Abstract

Atopic dermatitis (AD) is a chronic inflammatory skin disease characterized by epidermal barrier dysfunction, immune dysregulation, and marked clinical heterogeneity. Growing evidence implicates the gut microbiome in AD-related pathways through microbial metabolites, intestinal barrier function, and systemic immune signaling. This narrative review synthesizes current evidence on gut microbial alterations in AD, with particular attention to short-chain fatty acids, tryptophan-derived aryl hydrocarbon receptor ligands, intestinal permeability, gut–skin microbiome interactions, and microbiota-targeted interventions. Human studies have reported associations between AD and altered abundance of selected microbial taxa, metabolite profiles, and markers of intestinal barrier dysfunction, whereas animal and in vitro studies provide complementary mechanistic evidence. However, findings remain heterogeneous across age groups, disease phenotypes, geographic populations, analytical platforms, and treatment exposures, and causality is incompletely established. Probiotic and synbiotic interventions have shown strain-specific and context-dependent effects, while postbiotics, fecal microbiota transplantation, washed microbiota transplantation, and metabolite-directed approaches remain investigational. AI-assisted multi-omics methods may improve biological stratification and hypothesis generation, but current applications are limited by small sample sizes, cohort heterogeneity, overfitting, insufficient external validation, and limited clinical implementation. Current evidence therefore supports the gut microbiome as a mechanistically plausible contributor, potential biomarker, and therapeutic target in AD while underscoring the need for longitudinal, phenotype-aware, and externally validated studies before routine clinical translation.

## 1. Introduction

Atopic dermatitis (AD) is a chronic, relapsing inflammatory skin disease characterized by intense pruritus, erythematous lesions, and a profoundly compromised quality of life. Once regarded primarily as a pediatric condition, AD is now recognized as a persistent and increasingly prevalent disorder across all age groups. According to data from the Global Burden of Disease (GBD) consortium, at least 171 million individuals were affected worldwide in 2019, corresponding to approximately 2.23% of the global population [1]. More recent estimates place global prevalence at 11.1% among children and adolescents and 6.3% among adults, with the highest burden observed in Asia and in urban environments in low- to middle-income countries [2,3]. Beyond its epidemiological scope, AD imposes a substantial multidimensional burden: patients experience sleep disturbances, anxiety, depression, and social stigmatization, while healthcare systems face escalating direct and indirect costs estimated to exceed $5 billion annually in the United States alone [4,5].

The pathogenesis of AD is multifactorial and involves the interplay of genetic predisposition, epidermal barrier dysfunction, immune dysregulation, and environmental exposures. At its core, AD is driven by a Th2-polarized immune response characterized by elevated levels of interleukin (IL)-4, IL-5, IL-13, and IL-31, along with alarmin release (thymic stromal lymphopoietin [TSLP], IL-25, and IL-33) from damaged keratinocytes [6]. Loss-of-function mutations in the filaggrin gene (FLG), present in 10–30% of AD patients, exemplify the genetic basis for impaired skin barrier integrity, thereby facilitating allergen penetration, microbial colonization—particularly by *Staphylococcus aureus*—and perpetuating the inflammatory cycle [7]. In the chronic phase, Th22 and Th17 responses further contribute to epidermal thickening and barrier deterioration through IL-22, which suppresses filaggrin expression and antimicrobial peptide production [8].

Despite significant advances in targeted therapies—including dupilumab (anti-IL-4Rα), tralokinumab (anti-IL-13), and JAK inhibitors—a substantial proportion of patients continue to experience inadequately controlled disease, recurrence upon treatment discontinuation, and comorbidities extending beyond the skin into the atopic march (asthma, allergic rhinitis, food allergy) [9]. These limitations underscore the need for a broader mechanistic understanding of AD and for identifying novel therapeutic targets that operate upstream of or in parallel with the type 2 inflammatory cascade.

In recent years, increasing attention has been directed toward the gut microbiome as a critical yet underexplored modulator of AD pathogenesis. The “gut-skin axis”—a bidirectional biological conduit linking the intestinal and cutaneous microenvironments through immune, neuroendocrine, and metabolic pathways—has emerged as a conceptual framework of growing clinical relevance [10,11]. Notably, the skin and gut share fundamental structural and functional characteristics: both are primary interfaces between the host and the external environment; both are central components of mucosal immunity, and both are directly colonized by complex microbial communities whose composition profoundly influences systemic immune tone [12].

Epidemiological observations have long suggested a gut–skin relationship in AD: infants colonized by *Clostridium difficile* or *Escherichia coli* in early life, those born by cesarean section, and those exposed to antibiotics in the perinatal period demonstrate higher rates of subsequent AD development—all scenarios associated with gut microbiome perturbation [13]. Conversely, a growing number of studies have documented consistent patterns of gut dysbiosis in AD patients, including reduced microbial diversity, depletion of butyrate-producing taxa (*Faecalibacterium prausnitzii*, *Bifidobacterium*, *Blautia*, *Eubacterium*), and alterations in short-chain fatty acid (SCFA) and tryptophan metabolite profiles that correlate with disease severity [14,15].

Two dimensions of this relationship merit particular emphasis in current research. First, microbial metabolites—principally SCFAs (butyrate, propionate, acetate) and tryptophan-derived compounds (indoles, kynurenines)—function as key immunological mediators bridging the gut lumen with systemic and cutaneous immune responses, modulating regulatory T cell (Treg) differentiation, IL-10 and TGF-β production, and aryl hydrocarbon receptor (AhR) activation [16]. Second, the integration of multi-omics platforms (metagenomics, metabolomics, transcriptomics) with artificial intelligence and machine learning tools is beginning to reveal individualized microbial signatures with diagnostic and prognostic value, offering the prospect of precision medicine approaches to AD management [17]. Nevertheless, most human microbiome studies in AD remain observational, and reported associations should not be interpreted as proof that dysbiosis precedes or may contribute to disease. Reverse causation, treatment exposure, diet, age, and shared environmental determinants remain important alternative explanations.

Against this background, the present narrative review aims to synthesize the current evidence on the gut microbiome’s role in AD, with a dual focus on (i) the mechanistic underpinnings of the gut–skin axis, with particular attention to microbial metabolite pathways, and (ii) microbiota-targeted therapeutic strategies, from probiotic and prebiotic interventions to fecal microbiota transplantation (FMT). Additionally, we discuss the emerging contribution of AI-assisted multi-omics analysis in decoding the complexity of gut–skin interactions and its implications for future personalized therapeutic strategies. By integrating mechanistic insights with translational and clinical perspectives, this review aims to serve both dermatologists and researchers in microbiology and immunology who seek a comprehensive, up-to-date synthesis of this rapidly evolving field.

## 2. Literature Search Strategy and Evidence Synthesis

This narrative review was informed by a structured literature search conducted in PubMed. Earlier landmark studies were retained when necessary to provide mechanistic or historical context. Search terms were used individually and in combination and included ‘atopic dermatitis’, ‘eczema’, ‘gut microbiome’, ‘gut microbiota’, ‘gut-skin axis’, ‘dysbiosis’, ‘intestinal permeability’, ‘short-chain fatty acids’, ‘tryptophan metabolites’, ‘aryl hydrocarbon receptor’, ‘bile acids’, ‘probiotics’, ‘prebiotics’, ‘synbiotics’, ‘postbiotics’, ‘fecal microbiota transplantation’, ‘washed microbiota transplantation’, ‘metagenomics’, ‘metabolomics’, ‘multi-omics’, ‘machine learning’, and ‘artificial intelligence’.

Eligible publications included randomized and non-randomized clinical studies, prospective and retrospective observational studies, longitudinal cohorts, systematic reviews, meta-analyses, and experimental studies directly relevant to predefined thematic domains of gut microbial composition, microbial metabolites, intestinal barrier function, immune signaling, clinical severity, microbiome-targeted interventions, and multi-omics approaches in AD. Animal and in vitro studies were included when they addressed mechanisms not directly testable in humans and are explicitly identified as experimental evidence in the text.

Studies were excluded when they lacked direct relevance to AD or gut–skin axis biology, provided insufficient methodological information, duplicated previously reported cohorts without additional relevant analyses, or did not permit meaningful clinical or mechanistic interpretation. Reference lists of relevant primary studies and reviews were also screened to identify additional eligible publications.

Evidence was interpreted according to study design, sample size, population characteristics, replication across independent cohorts, consistency of findings, and directness of clinical relevance. Randomized trials, systematic reviews, and meta-analyses were prioritized for therapeutic interpretation, whereas observational studies were used primarily to assess associations and experimental studies to evaluate mechanistic plausibility.

## 3. The Gut–Skin Axis: Bidirectional Crosstalk in Atopic Dermatitis

### 3.1. Conceptual Framework and Structural Parallels

The gut–skin axis (GSA) describes a complex, bidirectional communication network linking the gastrointestinal tract with the skin through immune modulation, systemic inflammation, neuroendocrine signaling, and microbiota-derived metabolites [11,18,19]. Far from being a purely theoretical construct, this axis reflects genuine biological convergence: both the gastrointestinal tract and the skin are extensive epithelial interfaces that continuously interact with environmental antigens, microbial communities, and host immune networks. Modern morphometric estimates place the internal surface area of the adult human digestive tract at approximately 32 m^2^, substantially below the historically cited value of approximately 400 m^2^, which serves as a primary barrier between the host and the external environment. Both tissues are continuously exposed to antigens, allergens, and commensal microorganisms, and both play essential and overlapping roles in mucosal immunity [12,20].

At a structural level, the gut and skin share several key features. Both are regulated by tight junction (TJ) proteins—including occludin, claudins, and zonula occludens-1 (ZO-1)—that govern paracellular permeability and determine barrier selectivity [21]. In healthy conditions, these junctions prevent the translocation of microbial products and antigens into the systemic circulation. Both barriers are also rich in innate immune sentinels (dendritic cells, macrophages, mast cells) and adaptive immune effectors (Th1, Th2, Th17, and regulatory T cells [Tregs]), creating tissue-specific but interconnected immunological niches [14]. Although the epidermis and intestinal epithelium arise from distinct embryologic lineages, they share important functional characteristics as barrier organs, including regulated epithelial permeability, continuous microbial exposure, innate and adaptive immune surveillance, and communication with systemic immune and neuroendocrine networks [10].

### 3.2. Immune Crosstalk: Th1/Th2 Imbalance, IgE Dysregulation, and Treg Insufficiency

A pivotal mechanism through which the gut microbiome influences AD is via modulation of systemic immune balance. Under homeostatic conditions, a diverse and stable gut microbiota promotes the development of Tregs—both thymus-derived (tTregs) and peripherally induced (pTregs)—which suppress mucosal Th2-mediated inflammation, inhibit IgE class switching, and maintain immunological tolerance [15]. Gut-resident genera such as *Bifidobacterium*, *Clostridium*, *Bacteroides*, *Streptococcus*, and *Lactobacillus* produce short-chain fatty acids (SCFAs)—principally butyrate and propionate—that act as potent Treg inducers via histone deacetylase (HDAC) inhibition and GPR41/GPR43 receptor signaling [14,15].

In AD patients, gut dysbiosis disrupts this regulatory balance. Reduced microbial diversity and depletion of SCFA-producing taxa result in a shift toward unchecked Th2 polarization, with elevated production of IL-4, IL-5, and IL-13, and increased IgE synthesis by B cells [13]. Intestinal immune cells—particularly dendritic cells and Th17 cells—activated in the dysbiotic gut can migrate via systemic circulation to the skin and amplify local inflammation, a phenomenon documented in both AD and psoriasis [22,23]. This conceptual link between gut microbial poverty and allergic sensitization is central to the “hygiene hypothesis” and its more recent reformulation as the “old friends” hypothesis [24,25].

### 3.3. Intestinal Barrier Dysfunction and Increased Permeability

A critical component of gut–skin communication in AD is the integrity of the intestinal epithelial barrier. Compromised tight junction function may increase intestinal permeability and facilitate exposure of the lamina propria and systemic circulation to luminal microbial products, dietary antigens, and pathogen-associated molecular patterns. This process is more precisely described as intestinal barrier dysfunction or increased intestinal permeability rather than by the nonspecific term ‘leaky gut’ [26,27,28]. Zonulin, a physiological modulator of intestinal TJ permeability, has emerged as a measurable biomarker of gut barrier dysfunction. Elevated serum zonulin levels have been demonstrated in AD patients, particularly in children, and correlate independently with disease presence and severity, irrespective of total IgE and eosinophil counts [29]. Additional biomarkers include LBP, regenerating islet-derived protein 3 alpha (Reg3A), IL-10, and IL-22 [30]. This parallel impairment of intestinal and cutaneous TJs, both driven by Th2 cytokines, represents a mechanistic convergence reinforcing the notion of a systemic barrier disease [31,32,33,34].

### 3.4. Neuroendocrine Pathways: The HPA Axis, Cortisol, and the Itch-Stress Loop

Beyond immunological mechanisms, the gut–skin axis is connected through neuroendocrine signaling, most notably the hypothalamic–pituitary–adrenal (HPA) axis. HPA axis dysfunction documented in AD patients elevates systemic cortisol, paradoxically promoting Th2 skewing and suppressing antimicrobial immune responses [35]. Psychological stress activates the HPA axis via CRH and ACTH, simultaneously impairing gut barrier integrity and propagating downstream cutaneous inflammation [36]. Tryptophan, as the primary serotonin precursor, influences central serotonin concentrations and itch perception in AD independently of immune mechanisms, while SCFAs stimulate the sympathetic nervous system to modulate cutaneous lesion phenotype through neuronal pathways [37,38,39].

### 3.5. A Systems View: The Bidirectionality of the Gut–Skin Axis in AD

The gut–skin axis in AD is best considered a potentially bidirectional network rather than a unidirectional causal chain. Skin barrier dysfunction, increased transepidermal water loss, *Staphylococcus aureus* colonization, and alarmin release may contribute to systemic immune signaling that could, in turn, influence intestinal permeability and microbial ecology. Experimental studies also suggest that cutaneous perturbations can affect distant mucosal responses. For example, mechanical skin injury has promoted intestinal mast-cell expansion in experimental models, while ultraviolet B exposure has been associated with changes in intestinal microbial composition. The extent to which these observations translate into clinically meaningful bidirectional signaling in human AD remains uncertain [22,40].

**Figure 1:**
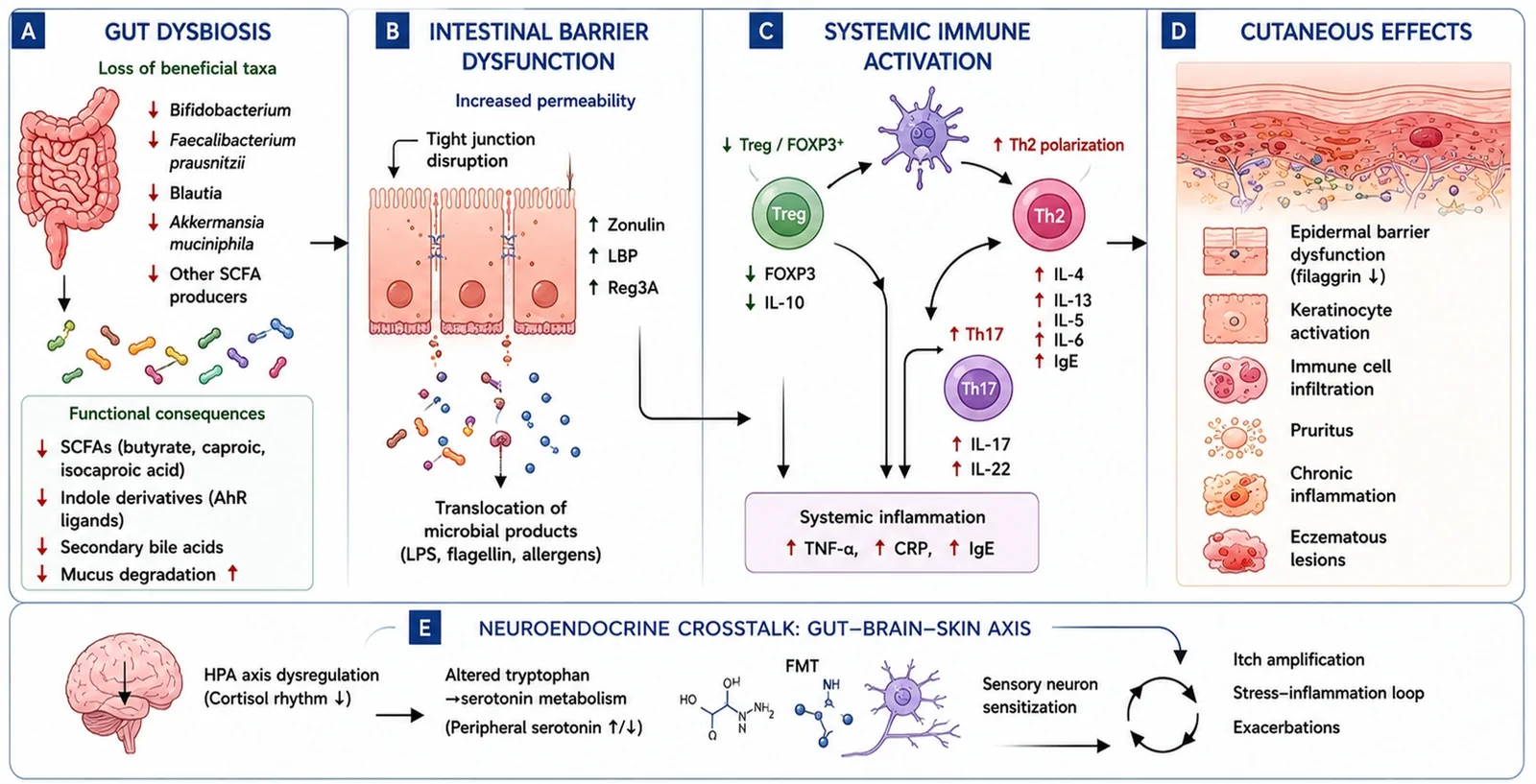
**The Gut–Skin Axis in Atopic Dermatitis: From Intestinal Dysbiosis to Cutaneous Inflammation. (Figure created in Canva).** Gut dysbiosis in AD is characterized by reduced abundance of beneficial microbial taxa, including *Bifidobacterium*, *Faecalibacterium prausnitzii*, *Akkermansia muciniphila*, and *Blautia*, alongside expansion of pathobionts. Loss of SCFA- and indole-producing bacteria impairs intestinal epithelial integrity, increasing permeability and the systemic translocation of microbial products, including lipopolysaccharide (LPS), flagellin, and allergens. Barrier dysfunction is associated with elevated zonulin, lipopolysaccharide-binding protein (LBP), and Reg3A. Systemic immune activation has been associated with reduced FOXP3+ regulatory T cells (Tregs), exaggerated Th2/Th17 polarization, elevated IgE production, and increased levels of inflammatory cytokines. Cutaneous consequences include keratinocyte activation, epidermal barrier disruption, pruritus, and chronic eczema lesions. Neuroendocrine pathways involving hypothalamic–pituitary–adrenal (HPA) axis dysregulation and altered tryptophan-serotonin metabolism further amplify the gut–brain–skin inflammatory cycle. Arrow symbols: ↑ indicates increased levels, activation, expression, abundance, or disease severity; ↓ indicates decreased levels, activation, expression, abundance, or disease severity; → indicates mechanistic or functional relationships; curved arrows indicate bidirectional interactions or feedback loops.

These proposed interactions are summarized in Figure 1, while their relative contribution to individual AD phenotypes and their causal direction remain uncertain.

### 3.6. Gut–Skin Microbiome Interactions in AD

The skin microbiome represents a complementary component of gut–skin axis biology in AD. Cutaneous flares are frequently associated with reduced microbial diversity and increased dominance of *Staphylococcus aureus*, although the magnitude and clinical relevance of these changes vary across body sites, age groups, disease severity, and treatment states. Epidermal barrier dysfunction, including filaggrin deficiency, can alter the local physicochemical environment and facilitate microbial imbalance. In parallel, type 2 cytokines, particularly IL-4 and IL-13, may impair selected antimicrobial and barrier-related responses, thereby increasing susceptibility to cutaneous dysbiosis. Gut and skin microbial alterations should not be interpreted as a single unified microbial process. Current evidence more strongly supports indirect communication through immune, metabolic, and barrier pathways than direct transfer of gut microorganisms to the skin. Age, diet, antibiotics, host genetics, disease severity, topical therapy, systemic treatment, and environmental exposure may influence both microbial compartments simultaneously and therefore represent important confounders. Paired longitudinal studies of gut and skin microbiomes are needed to determine whether changes in one compartment precede, follow, or merely accompany changes in the other [41,42,43,44].

## 4. Gut Microbiome Composition in Atopic Dermatitis

### 4.1. Compositional Dysbiosis as a Recurrent Feature Reported in AD

One of the most consistently replicated findings in gut microbiome research of AD is compositional dysbiosis—reduced alpha diversity, depletion of beneficial anaerobic taxa, and enrichment of potentially pathogenic microorganisms [13,14,15,45]. AD patients exhibit recurring dysbiotic patterns across diverse cohorts and age groups converging on a common theme: insufficiency of SCFA-producing, immunoregulatory bacteria and compensatory overgrowth of taxa associated with barrier disruption and Th2 skewing [46,47].

### 4.2. Depletion of Beneficial Taxa: The Immunoregulatory Deficit

Taxonomic findings should be interpreted cautiously because no single microbial signature has been consistently reproduced across all AD populations. Differences in age, geography, diet, treatment exposure, sequencing platform, and disease phenotype may account for part of the observed variability.

The most clinically significant finding in AD gut dysbiosis is the consistent reduction in immunoprotective genera:

• *Bifidobacterium* spp.—the most consistently depleted genus in AD; reductions correlated with disease severity and acute-phase flares; their depletion was associated with subsequent persistent eczema trajectories [15,48].
• *Faecalibacterium prausnitzii*—a keystone anti-inflammatory butyrate producer; its loss destabilizes gut epithelial integrity and is associated with skin microbiome perturbations [15,16].
• *Blautia*, *Coprococcus eutactus*, and *Eubacterium* spp.—SCFA-producing Lachnospiraceae members whose reduction correlates with diminished butyrate output, more pronounced in severe AD [49].
• *Akkermansia muciniphila*—absent in AD infants and their mothers; its presence after vaginal delivery protects against AD onset; reduced colonization correlates with impaired PI3K-Akt and immune development gene expression [50,51].
• *Lactobacillus* spp.—broadly reduced in AD; associated with increased flare frequency and disease severity [46].

### 4.3. Enrichment of Potentially Pathogenic Taxa

The gut microbiome of AD patients is enriched for pro-inflammatory taxa: early colonization with *Clostridium difficile* and *Escherichia coli* was associated with subsequent eczema development by dysregulating Treg induction; *Klebsiella* spp. and *E. coli* show significant overabundance (*p* < 0.05) in AD infants [52]; shifts in the *Firmicutes*/*Bacteroidetes* ratio are observed in both human and murine AD models [53]; and *Ruminococcus gnavus* enrichment shows context-dependent dysbiotic roles in AD subgroups [51]. In adult AD, *Bacteroidales*, *Enterobacteriaceae*, and *Clostridium perfringens* are more characteristic of the fecal microbiota compared to healthy adults [46].

### 4.4. Pediatric vs. Adult Gut Microbiome Profiles

#### 4.4.1. Infant and Early Childhood AD

The first three years of life are a critical window of gut microbiome establishment [54]. In infants with AD, dysbiosis includes reduced *Bifidobacterium* richness from 4–26 weeks postpartum, increased *Parabacteroides* and *Enterobacteriaceae*, and disordered temporal colonization by SCFA-producing taxa—leading to butyrate deficiency and microaerophilic destabilization of the anaerobic microbiome [55]. Dietary patterns significantly modulate severity: processed-food-dominant diets are associated with higher abundances of Dorea and Anaerostipes and worse EASI/SCORAD scores [56,57].

#### 4.4.2. Adult AD

In adult AD, alpha diversity may be preserved while functional SCFA deficit persists [46]. Enrichment of *Enterobacteriaceae* and reduced abundance of *Bifidobacterium* and *Lactobacillus* have also been reported during remission in selected cohorts, suggesting that some microbial alterations may persist beyond clinically apparent flares; however, longitudinal evidence establishing temporal precedence remains limited [47].

### 4.5. AD Phenotypes and Endotypes as Sources of Microbiome Heterogeneity

AD should not be treated as a microbiologically uniform disease. Pediatric and adult AD differ in immune maturation, dietary exposure, medication use, and microbiome stability, while intrinsic and extrinsic forms differ in patterns of systemic allergic sensitization and IgE expression. FLG status, food allergy, atopic march trajectories, severe or refractory disease, and ethnicity-associated immune endotypes may further modify host–microbiome relationships. These variables may partly explain inconsistent taxonomic findings across cohorts and should be incorporated into future microbiome study designs and stratified analyses. A microbial signature identified in infants at risk of AD should not be assumed to apply to adults with longstanding moderate-to-severe disease. Similarly, associations observed in IgE-high extrinsic AD may not generalize to IgE-normal or intrinsic phenotypes. Treatment exposure is another major source of heterogeneity because topical anti-inflammatory therapy, systemic immunomodulators, biologics, JAK inhibitors, antibiotics, and dietary restriction may alter host immunity, microbial composition, or both. Future studies should therefore report and, where possible, stratify analyses by age, disease severity, phenotype, FLG status, allergic comorbidity, ethnicity, and treatment exposure [58,59,60,61,62].

### 4.6. Potential Biomarker Taxa

*Bacteroidaceae* and *Porphyromonadaceae* have been identified as potential fecal biomarker families for AD diagnosis [16]. Baseline gut microbiome composition has been investigated as a predictor of response to the specific probiotic formulation evaluated in the cited study, with responders showing enrichment of selected microbial taxa (*Clostridium*, *Lactobacillus*, and *Streptococcus*) [63]. The gut microbiota is being investigated as a potential early biomarker, treatment-response indicator, and potential therapeutic target in AD, although none of these roles is currently established for routine clinical use [14].

### 4.7. Methodological Considerations

16S rRNA amplicon sequencing remains the predominant platform but is limited in species-level resolution and functional gene content. Whole-metagenome sequencing (WMS) provides species-level resolution and direct functional gene annotation—essential for capturing SCFA biosynthesis, vitamin production, and immune signaling dysbiosis not visible in compositional profiles alone [51,56]. Methodological standardization—uniform sequencing depth, bioinformatic pipelines, fecal sample handling—remains a significant unmet need.

Cross-study comparability is further limited by differences in DNA extraction, sequencing region, sequencing depth, taxonomic database, compositional data handling, adjustment for multiple testing, and control of clinical confounders. Stool samples also provide an incomplete representation of mucosa-associated microbial communities. These methodological differences should be considered when interpreting apparently conflicting taxonomic associations.

## 5. Microbial Metabolites as Key Mediators in the Gut–Skin Axis

**Figure 2:**
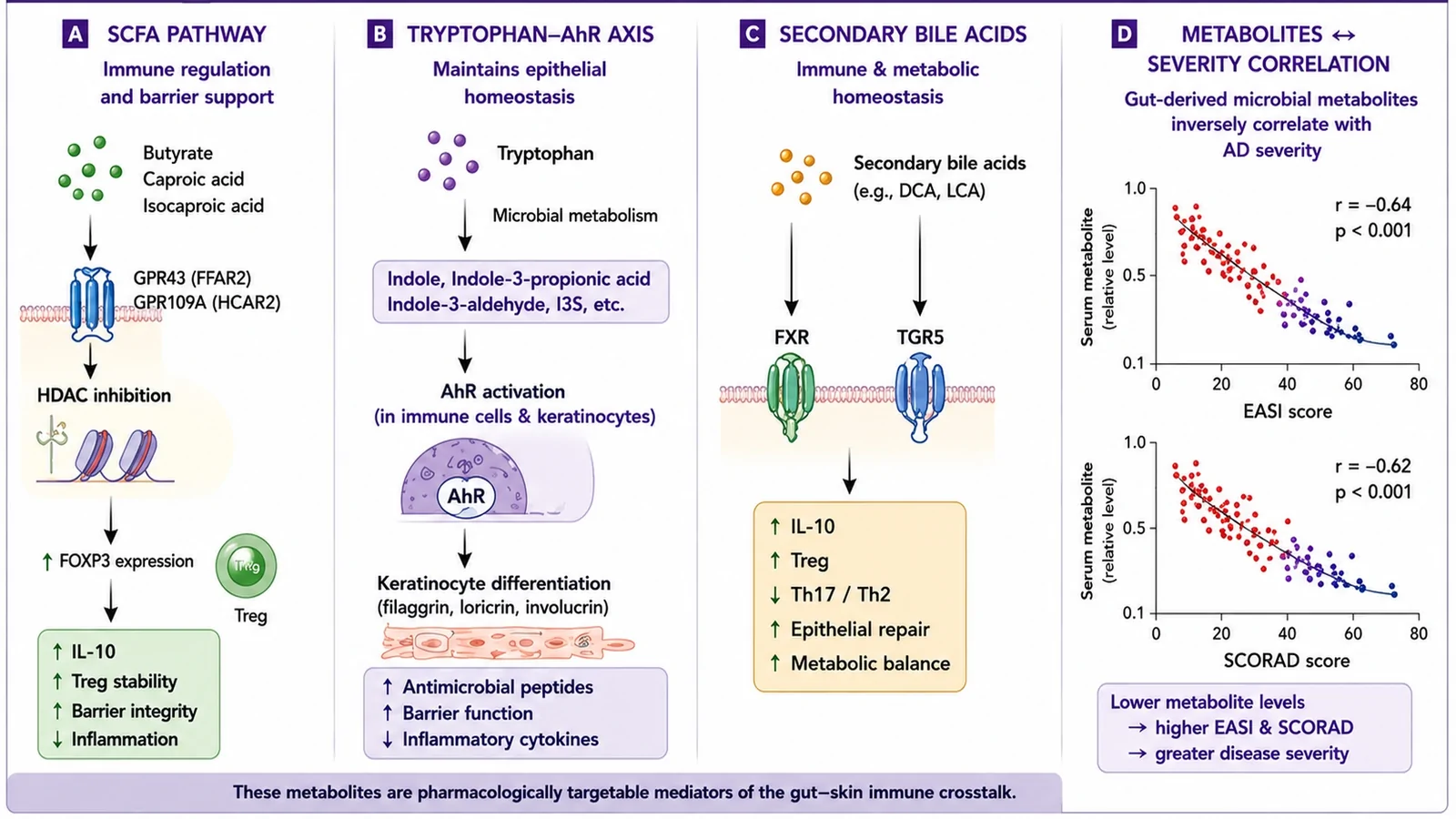
**Microbial metabolites as key immunologic mediators in atopic dermatitis (Figure created in Canva).** SCFAs including butyrate, caproic acid, and isocaproic acid activate GPR43 (FFAR2) and GPR109A signaling pathways, inhibit histone deacetylases (HDACs), and promote FOXP3+ Treg differentiation, IL-10 production, epithelial barrier integrity, and suppression of inflammation. Tryptophan-derived indole metabolites activate the aryl hydrocarbon receptor (AhR) in immune cells and keratinocytes, enhancing epidermal differentiation, antimicrobial peptide production, and immune tolerance while reducing pro-inflammatory cytokine signaling. Secondary bile acids regulate epithelial and immune homeostasis through activation of the FXR and TGR5 receptors. Reduced circulating levels of microbial metabolites correlate inversely with EASI and SCORAD scores and positively with inflammatory biomarkers, supporting their role as mechanistic drivers and candidate biomarkers of AD severity [36]. Arrow symbols: ↑ indicates increased levels, activation, expression, abundance, or disease severity; ↓ indicates decreased levels, activation, expression, abundance, or disease severity; → indicates mechanistic or functional relationships.

The gut microbiome exerts much of its immunological influence through bioactive metabolites that enter systemic circulation and reach distant organs, including the skin. Among the most extensively studied are SCFAs, tryptophan-derived metabolites, and secondary bile acids—whose production is critically dependent on microbial diversity and whose depletion in AD patients correlates closely with disease activity. The evidence base differs substantially across metabolite classes. Human observational studies primarily support associations between circulating or fecal metabolite concentrations and disease activity, whereas many receptor-specific and downstream immune mechanisms derive from animal models, ex vivo systems, or in vitro experiments. These evidence levels are distinguished throughout the following subsections [14,16]. Figure 2 summarizes selected mechanistic pathways through which microbial metabolites may influence host–microbiome communication.

### 5.1. Short-Chain Fatty Acids (SCFAs): Immunoregulatory Keystones

#### 5.1.1. Treg Induction and Th2 Suppression

Mechanistic evidence, derived largely from experimental, ex vivo, and in vitro systems, indicates that SCFAs can influence immune regulation through G protein-coupled receptor signaling and histone deacetylase inhibition. SCFAs—butyrate, propionate, and acetate—are produced by anaerobic fermentation of dietary fiber by gut commensals, including *Faecalibacterium prausnitzii*, *Bifidobacterium*, *Blautia*, *Roseburia*, and *Coprococcus* spp. [15]. They act through two major molecular mechanisms: (i) activation of GPCRs—GPR41, GPR43, and GPR109A; and (ii) inhibition of histone deacetylases (HDACs), driving epigenetic remodeling that promotes immune tolerance [64,65]. Butyrate enhances histone H3 acetylation at the Foxp3 locus in CD4+ T cells, amplifying Treg generation [65,66]. Propionate exerts analogous effects, while acetate acts through GPR43 to suppress neutrophil-mediated inflammation [64]. SCFA depletion in AD results in reduced FOXP3+ Treg activity, unopposed Th2 polarization, and elevated IgE [67].

#### 5.1.2. Clinical Correlations: SCFAs and AD Severity

A case–control study of 50 adult AD patients and 25 controls found that serum concentrations of caproic acid and isocaproic acid were strongly negatively correlated with EASI and SCORAD (r = −0.33 to −0.37, *p* < 0.05) [68]. Additional SCFAs, such as propionic acid, butyric acid, and valeric acid, are also inversely correlated with disease extent during remission [30]. A longitudinal infant cohort confirmed significant negative associations between plasma concentrations of caproic, acetic, and succinic acids and the subsequent development of atopic sensitization and eczema [69].

### 5.2. Tryptophan Metabolites and the Aryl Hydrocarbon Receptor (AhR) Pathway

#### 5.2.1. AhR Activation: Barrier Reinforcement and Anti-Inflammatory Signaling

Tryptophan catabolism by intestinal microbiota generates indole derivatives—indole-3-aldehyde (IAld), indole-3-acetic acid (IAA), indole-3-propionic acid (IPA), indole-3-lactic acid (I3LA), and tryptamine—which function as AhR ligands [16,53,70,71]. AhR activation in keratinocytes upregulates filaggrin and barrier proteins, reduces TSLP expression, and promotes antimicrobial peptide production. IAld levels are significantly lower in both lesional and non-lesional skin of AD patients; topical IAld application in a murine AD model significantly attenuated skin inflammation, inhibited Th2 cytokines and TSLP—effects abolished in AhR-null mice and by AhR antagonist treatment [72]. I3LA treatment of IL-4/IL-13-stimulated human skin equivalents restored barrier protein expression and reduced AD-associated gene expression [70].

#### 5.2.2. AhR Dysregulation in AD

In AD, multiple converging factors deplete AhR signaling: gut dysbiosis reduces tryptophan catabolism; Th2 inflammation suppresses AhR target gene expression (CYP1A1 is significantly reduced in lesional skin by scRNA-seq); and accumulation of toxic indoxyl sulfate disrupts skin vascular homeostasis and worsens neuroinflammation and pruritus [14,73].

### 5.3. Secondary Bile Acids: Emerging Modulators of Barrier and Immunity

Secondary bile acids, lithocholic acid (LCA) and omega-muricholic acid (omega-MCA), modulate immunity via the farnesoid X receptor (FXR) and TGR5. Patients with AD exhibit reduced omega-MCA levels, thereby weakening FXR signaling and impairing Treg function [14]. LCA and omega-MCA enhance AMP expression in the skin and improve intestinal barrier function. In murine AD models, bile acid supplementation reduced epidermal thickening and cutaneous inflammatory cytokines—though this has not yet been validated in human trials [14,74]. These findings remain predominantly preclinical and have not yet been confirmed in adequately powered human interventional studies of AD.

### 5.4. Other Bioactive Metabolites

TMAO is reduced in AD patients and may modulate systemic inflammatory tone [30]. Linoleic acid and its microbially modified derivatives alleviate AD-like symptoms in mouse models through lipid-microbiome crosstalk. Polyamines produced by gut bacteria support intestinal epithelial renewal with emerging immunomodulatory relevance in AD [74].

### 5.5. Metabolomics as a Translational Bridge

The metabolite-centric perspective provides mechanistically interpretable, clinically measurable endpoints correlatable with EASI/SCORAD and potentially leverageable for patient stratification. Integration of metabolomics data with multi-omics platforms is beginning to reveal individualized gut–skin metabolite “fingerprints” in AD—the foundation for AI-assisted precision medicine strategies discussed in Section 6.

Several gut-derived metabolites and intestinal barrier-associated markers have been associated with AD severity, although their clinical validity remains preliminary and study-dependent. Representative findings are summarized in Table 1.

## 6. Artificial Intelligence and Multi-Omics Integration in Decoding the Gut–Skin Axis

### 6.1. The Imperative for Integrative Approaches

The gut–skin axis in AD is a multidimensional biological system not amenable to characterization by a single analytical layer. Classical single-omics approaches provide valuable but inherently incomplete snapshots [75,76,77,78,79]. The proliferation of multi-omics platforms simultaneously interrogating metagenomics, metabolomics, transcriptomics, proteomics, epigenomics, and lipidomics—combined with AI/ML capable of identifying complex, non-linear patterns in high-dimensional datasets—is beginning to reveal previously unrecognized links between microbial metabolic pathways and host immune signaling networks [16,80,81].

### 6.2. Multi-Omics Platforms: Layering the Biological Architecture of AD

#### 6.2.1. From Single to Integrated Omics

A landmark multi-omics study employing microbiota analysis, metabolomics, and intestinal epithelial transcriptomics across 2247 children from the COCOA birth cohort linked early-life gut microbial composition to metabolite-driven epigenetic modifications associated with subsequent AD development [16]. Multi-omics overlap analyses confirm that filaggrin (FLG) is the only candidate consistently implicated across genomic, epigenomic, transcriptomic, and proteomic layers, underscoring the value of integrated analysis [75].

#### 6.2.2. Endotype Discovery Through Multi-Omics Integration

Integrated metagenomics, metabolomics, and single-cell transcriptomics identified two endotype-specific gut–skin axis signatures: a Bacteroides-enriched enterotype associated with LPS-driven systemic inflammation and impaired barrier function, and a Prevotella-dominant cluster linked to enhanced AhR activation and epithelial-protective metabolite profiles [82]. Single-cell RNA sequencing and spatial transcriptomics identified unique inflammatory fibroblast subpopulations (COL18A1+) and complex intercellular signaling networks not visible with bulk transcriptomics [83]. scRNA-seq of Langerhans cells from AD patients revealed altered transcriptional profiles that bridge *S. aureus* recognition and T cell activation [84].

### 6.3. Machine Learning in Gut Microbiome Analysis

#### 6.3.1. Interpretable ML Models for AD-Associated Gut Features

Ma et al. constructed an interpretable ML framework applying five algorithms—random forest (RF), LightGBM, XGBoost, support vector machine, and logistic regression—to 16S rRNA datasets after centered log-ratio (CLR) transformation. RF achieved the highest discriminatory performance. SHAP values quantified each taxon’s marginal contribution to AD risk, identifying *Bifidobacterium* and *Collinsella* as among the most predictively significant features, with partial dependence plots characterizing their non-linear influence on AD risk [80].

#### 6.3.2. Graph Neural Networks and Deep Learning

ATOMIC, a graph attention network for AD prediction using gut microbiome data, demonstrated that modeling the relational structure of microbial co-occurrence networks substantially improved predictive accuracy over feature-based classifiers, capturing ecological dependencies within the gut community invisible to standard ML models [85].

#### 6.3.3. Mendelian Randomization and Causal Inference

A two-sample MR study using GWAS data from 207 gut microbial taxa (Dutch Microbiome Project, n = 7738) and AD outcome data (FinnGen biobank, 5321 AD patients, 213,146 controls) identified 17 distinct bacterial taxa—spanning 2 orders, 4 families, 5 genera, and 6 species—with statistically supported causal associations with AD risk [86]. These findings provide genetic-level evidence for the biological plausibility of the gut–skin axis. Mendelian randomization can strengthen causal inference but does not, by itself, establish causality. Interpretation depends on instrument strength, absence of horizontal pleiotropy, population comparability, and the validity of the underlying genetic assumptions.

### 6.4. AI-Assisted Multi-Omics for Precision Medicine in AD

Metabolomic profiling has identified alterations in SCFA and tryptophan metabolites that correlate with disease severity, while transcriptomic analyses link these metabolite changes to downstream gene expression networks in keratinocytes and immune cells [82]. AI-assisted multi-omics models are being developed to predict individual responses to microbiota-targeted interventions based on baseline gut microbiome and metabolite profiles—the most rapidly growing frontier in AD–microbiome research [16]. Key limitations include small sample sizes, absence of standardized protocols, limited racial diversity, and interpretability challenges in deep learning models [75,76,80].

Despite their analytical potential, AI-assisted microbiome models remain primarily research tools. Most available studies are limited by modest sample sizes relative to feature dimensionality, batch effects across sequencing and omics platforms, population heterogeneity, incomplete adjustment for clinical confounders, and limited external validation. Model performance may be inflated by overfitting, data leakage, or cohort-specific microbial patterns that do not generalize across populations. Interpretability also remains incomplete, and few models have undergone prospective evaluation or demonstrated added clinical value beyond established patient characteristics and severity measures [87,88,89,90].

### 6.5. Future Directions: Toward AI-Driven Precision Microbiome Medicine

The convergence of AI and multi-omics outlines a trajectory toward “precision microbiome medicine”—individualized microbiota-targeted interventions matched to specific gut–skin metabolite fingerprints and endotype classifications. Research priorities include multicenter longitudinal cohort studies with harmonized multi-omics protocols, validation of endotype signatures in prospective clinical trials, integration of AI decision-support tools into clinical workflows, and causal inference frameworks to move from association to mechanism [91].

**Figure 3:**
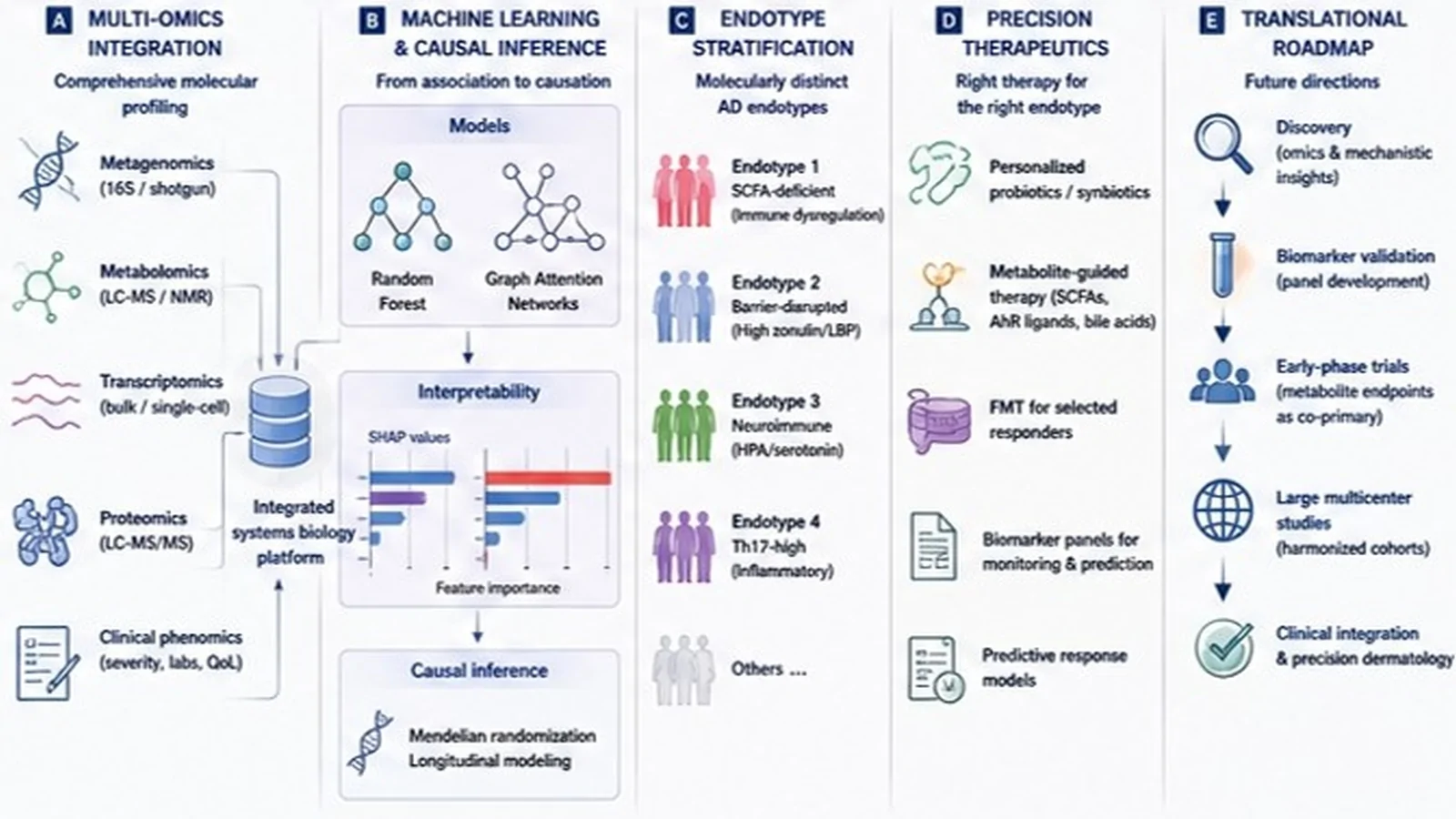
**AI-powered multi-omics integration and personalized medicine approaches in atopic dermatitis (created in Canva).** These platforms combine metagenomic, metabolomic, transcriptomic, proteomic, and single-cell data to study host–microbiome interactions in AD. Machine learning methods such as random forests, graph attention networks, SHAP explainability, and causal inference help identify key microbial factors, metabolite markers, inflammatory pathways, and predictive biomarkers. Multi-omics analysis allows for stratifying different AD endotypes, like SCFA-deficient, barrier-disrupted, neuroimmune, and Th17-high inflammatory types. These strategies support precision treatments through personalized probiotics, metabolite-based therapies, biomarker panels, response prediction models, and tailored microbiome interventions. Integrating AI and systems biology aims to advance biomarker validation, optimize clinical trials, and implement precision dermatology. This process requires validation within and outside the study, prospective testing, and proof of clinical usefulness. Current challenges involve small sample sizes, high data complexity, batch effects, cohort variability, overfitting risks, limited interpretability, and few implementation studies. The figure does not indicate that these methods are ready for routine clinical use.

Figure 3 summarizes potential research applications of AI-assisted multi-omics while also indicating the validation steps required before clinical implementation.

## 7. Therapeutic Strategies Targeting the Gut Microbiome in Atopic Dermatitis

### 7.1. Rationale for Microbiome-Targeted Therapies

Microbiome-targeted interventions should be interpreted in light of clinical purpose, population, formulation, disease severity, and evidence maturity. Prevention of incident AD is distinct from the treatment of established disease, and results from prenatal, infant, pediatric, and adult populations should not be conceptually pooled without acknowledging major biological and clinical differences. Probiotic effects are strain-specific and formulation-specific, and efficacy observed for one product should not be generalized to an entire genus or intervention class. Microbiome-targeted interventions aim to restore the ecological and metabolic conditions that underpin immune homeostasis—addressing upstream potential contributors of AD rather than its symptomatic manifestations [92,93,94]. This section reviews the principal therapeutic approaches: probiotics, prebiotics, postbiotics, synbiotics, and fecal microbiota transplantation (FMT).

### 7.2. Probiotics: Mechanisms, Strains, and Clinical Evidence

#### 7.2.1. Immunological Mechanisms of Action

Probiotics modulate AD through multiple mechanisms: increasing secretory IgA and suppressing IgE-mediated sensitization; promoting Treg differentiation and IL-10/ TGF-β production; enhancing tight junction expression; producing SCFAs that reduce intestinal permeability; and competing with S. aureus colonization through antimicrobial peptide secretion [92,95,96].

#### 7.2.2. Clinical Efficacy: Strain-Specific Evidence

A systematic review of 32 RCTs (2005) identified L. paracasei K71, L. plantarum IS-10506, and L. acidophilus L-92 as strains with the most consistent evidence for moderately reducing SCORAD and EASI. L. fermentum showed significant SCORAD reductions (MD = −11.42, 95% CI: −13.81 to −9.04); L. salivarius showed similarly significant effects (MD = −7.21, 95% CI: −9.63 to −4.78), while L. rhamnosus GG showed no significant effect (MD = 3.29, *p* = 0.07) [97,98]. A meta-analysis of 25 pediatric RCTs (Hedges’ g = 0.65, *p* < 0.05) indicated moderate-to-large improvement in AD severity [99].

#### 7.2.3. Timing: Prenatal vs. Postnatal

Combined prenatal and postnatal probiotic strategies confer the greatest preventive benefit, particularly in high-risk infants. The EAACI 2024 task force meta-analysis confirmed significant reductions in SCORAD with probiotics alone or in combination with prebiotics, along with additional improvements in patient-reported itch and sleep outcomes [93].

### 7.3. Prebiotics, Postbiotics, and Synbiotics

Prebiotics (FOS, GOS, HMOs) show more consistent benefit in AD prevention than treatment, with the 2025 umbrella meta-analysis confirming no significant SCORAD reduction from prebiotic-only interventions [100]. Postbiotics—preparations of inanimate microorganisms and/or their components—offer a heat-stable, bacteremia-free alternative that activates pattern recognition receptors and promotes Treg responses, currently limited to pilot studies [101,102]. Synbiotics demonstrated the most consistent SCORAD reductions across the subgroups analyzed in the 2025 umbrella meta-analysis, as the prebiotic component enhances probiotic colonization and the durability of effect [100].

### 7.4. Fecal Microbiota Transplantation (FMT)

#### 7.4.1. Rationale and Approaches

FMT enables comprehensive restoration of gut microbial ecology, aiming to reset the dysbiotic gut ecosystem toward an SCFA-producing, immune-tolerant composition [103,104]. Washed microbiota transplantation (WMT) incorporates additional processing steps intended to improve standardization and potentially reduce selected procedural risks; however, comparative safety and long-term clinical benefit in AD remain insufficiently established [105].

#### 7.4.2. Clinical Trial Evidence

FMT and WMT should currently be regarded as investigational interventions in AD. Preliminary human studies provide signals of potential biological and clinical activity, but the evidence base remains limited by small samples, limited replication, heterogeneous procedures, and insufficient long-term follow-up. These findings do not establish routine clinical utility. A randomized, double-blind, placebo-controlled trial evaluated FMT in 63 adult patients with moderate-to-severe AD: EASI scores and EASI-50 response rates were significantly higher in the FMT group. FMT decreased Th2/Th17 cell proportions, reduced serum TNF-α and total IgE, and produced significant shifts in gut microbial composition. No serious adverse reactions occurred [104]. WMT in 23 moderate-to-severe AD patients demonstrated significant decreases in SCORAD, EASI, DLQI, and NRS itch scores (all *p* < 0.05), with a reduction in peripheral basophils, increased gut microbial diversity, enrichment of *Bifidobacterium* and *Faecalibacterium*, and metabolomic shifts in SCFAs and bile acid derivatives [16,105].

Larger independent randomized trials, standardized processing protocols, long-term surveillance, and comprehensive safety reporting are required before FMT or WMT can be considered for routine AD care.

#### 7.4.3. Particularities of FMT/WMT

Safety considerations are particularly important because FMT and WMT involve transfer of complex, incompletely characterized biological material. Donor screening should include detailed medical, behavioral, travel, medication, and infectious-risk assessment, together with blood and stool testing for enteric pathogens, blood-borne infections, and multidrug-resistant organisms, including extended-spectrum beta-lactamase-producing Enterobacteriaceae, vancomycin-resistant enterococci, carbapenem-resistant Enterobacteriaceae, and methicillin-resistant Staphylococcus aureus. Serious infections due to pathogen or multidrug-resistant organism transmission have been reported after investigational FMT, including in immunocompromised recipients, prompting regulatory safety alerts and strengthened donor-screening requirements [106,107].

Pediatric patients and immunocompromised individuals require additional caution because immune immaturity, comorbid disease, concurrent immunosuppression, and altered barrier function may increase vulnerability to infectious or dysbiotic complications [108,109].

In addition, the long-term ecological and metabolic consequences of microbiota transfer remain incompletely defined, and regulatory frameworks for FMT, WMT, and related microbiota-based products differ across jurisdictions. Current guidance supports microbiota-based therapies mainly for selected Clostridioides difficile infection indications, whereas use in non-CDI conditions, including AD, should remain investigational and restricted to controlled clinical studies with standardized protocols, informed consent, adverse-event surveillance, and long-term follow-up [109,110].

### 7.5. Dietary Modulation

High dietary fiber intake and Mediterranean dietary patterns are associated with greater gut microbial diversity and lower levels of systemic inflammatory markers relevant to AD [111]. Processed-food-dominant diets in pediatric AD are associated with worse EASI/SCORAD scores [57]. Breastfeeding—the most potent dietary modulator of early microbiome composition—consistently demonstrates protective effects against AD development through HMO-mediated *Bifidobacterium* enrichment in the neonatal gut [111].

## 8. Discussion

### 8.1. Positioning This Review Within the Existing Literature

Most published reviews on the gut microbiome in AD focus either on compositional dysbiosis or on microbiota-targeted interventions, while few integrate mechanistic microbial metabolite pathways with emerging computational approaches. Furthermore, the rapidly expanding fields of AI, machine learning, and multi-omics analysis have rarely been discussed within a unified gut–skin axis framework. The present review addresses these gaps by integrating microbiome composition, metabolite-mediated immune regulation, microbiota-targeted therapeutics, and AI-assisted precision medicine into a single translational model of AD pathogenesis and management.

### 8.2. From Dysbiosis to Clinical Severity: A Quantitative Framework

A major conceptual contribution of this review is the integration of three interconnected biological layers: gut microbial composition, metabolite production, and clinical disease severity. Depletion of beneficial taxa such as *Bifidobacterium*, *Faecalibacterium prausnitzii*, *Blautia*, and *Akkermansia muciniphila* is consistently associated with reduced production of SCFA and tryptophan-derived metabolites, while altered concentrations of caproic acid, isocaproic acid, butyrate, indoxyl, and biomarkers of intestinal permeability correlate with EASI and SCORAD scores [30]. This integrated dysbiosis–metabolite–severity axis provides a biologically plausible framework for developing composite biomarker panels that combine microbial metabolites with markers of intestinal barrier dysfunction and systemic inflammation.

### 8.3. Microbial Metabolites as Therapeutic Targets

The evidence reviewed supports a transition from viewing microbial metabolites as passive biomarkers to considering them as active therapeutic targets. SCFAs regulate immune tolerance through HDAC inhibition, FOXP3 induction, and GPR43/GPR109A signaling, whereas tryptophan-derived metabolites modulate keratinocyte differentiation and immune homeostasis through AhR activation [14,65,67,72]. Similarly, secondary bile acids influence epithelial integrity and Treg function through FXR and TGR5 signaling pathways [67]. These findings provide a mechanistic rationale for targeted microbiome interventions designed to restore metabolite production rather than simply modify microbial composition.

### 8.4. AI and Multi-Omics as the Foundation of Precision Microbiome Medicine

Recent advances in AI and multi-omics technologies are transforming gut microbiome research from descriptive association studies toward clinically actionable precision medicine. Interpretable machine learning approaches, including SHAP-based models and graph neural networks, enable identification of key microbial taxa and metabolite signatures associated with AD risk and treatment response [16,80,82]. In parallel, Mendelian randomization studies provide increasing support for causal microbiome–AD relationships [86]. The integration of metagenomics, metabolomics, transcriptomics, and single-cell analyses is beginning to reveal biologically distinct AD endotypes that may ultimately guide personalized therapeutic selection and disease monitoring.

### 8.5. Clinical Applicability and Therapeutic Readiness

The clinical applicability of microbiome findings in AD remains limited. No gut microbial taxon, metabolite profile, or AI-derived signature has yet achieved sufficient prospective validation, reproducibility, and incremental clinical utility for routine patient stratification. Similarly, therapeutic evidence should be interpreted in light of intervention specificity and maturity. Selected probiotics and synbiotics have the largest clinical evidence base but remain heterogeneous, while FMT, WMT, postbiotics, and metabolite-directed strategies are investigational. Clinical translation will require standardized protocols, predefined clinically meaningful outcomes, independent replication, transparent safety reporting, and demonstration that microbiome-informed strategies improve outcomes beyond established clinical assessment and treatment pathways.

### 8.6. Current Challenges and Research Gaps

Despite significant progress, several important limitations remain. Microbiome studies continue to be affected by methodological heterogeneity, including differences in sequencing platforms, bioinformatic pipelines, and sample-processing procedures [100]. Geographic and ethnic representation remains limited, with most available datasets originating from East Asian and European populations [104]. Furthermore, although growing evidence supports causal links between gut dysbiosis and AD, many observations remain associative and require validation in longitudinal and interventional studies [1,62]. Additional uncertainties include interactions between microbiome-targeted therapies and established treatments, such as biologics and JAK inhibitors, as well as regulatory and safety considerations for FMT and live biotherapeutic products [44,87,112,113].

### 8.7. Causality, Reverse Causation, and Confounding

Interpretation of the AD microbiome literature is limited by substantial clinical and methodological heterogeneity. Reported microbial associations vary according to age, geographic region, diet, breastfeeding history, birth mode, antibiotic exposure, disease severity, allergic comorbidities, FLG status, treatment exposure, sequencing platform, sample handling, and bioinformatic pipeline. These variables can influence microbial composition independently of AD and may partly explain inconsistent taxonomic findings across cohorts [114,115].

Confounding is a major concern because many determinants of AD are also determinants of microbiome composition. Diet, socioeconomic context, urbanization, antibiotic use, mode of delivery, breastfeeding, ethnicity, food allergy, obesity, and medication exposure may influence both disease phenotype and microbial structure. Failure to measure or adequately adjust for these factors can generate associations that are difficult to interpret biologically [116,117].

Reverse causation represents an additional limitation. Dietary restriction, sleep disturbance, systemic inflammation, recurrent antibiotic exposure, topical and systemic treatments, and behavioral changes associated with severe disease may alter the gut microbiome after AD is established. Consequently, cross-sectional studies cannot determine whether dysbiosis precedes disease onset, results from the disease state, reflects treatment exposure, or arises from shared environmental determinants [118,119].

Causal inference, therefore, remains incomplete. Longitudinal birth cohorts, intervention studies, Mendelian randomization, and experimental models provide complementary evidence, but each approach has important limitations. Experimental findings may not translate directly to human AD. Mendelian randomization depends on instrument validity and underlying assumptions, and small interventional studies may be vulnerable to population-specific effects, limited power, and poor reproducibility [120,121].

Future studies should combine prospective sampling, repeated microbiome measurements, standardized clinical phenotyping, detailed treatment documentation, dietary assessment, metabolomics, and independent external validation. Stratification by age, disease severity, AD phenotype, FLG status, allergic comorbidity, ethnicity, and treatment exposure will be necessary to determine whether reproducible microbiome signatures exist within clinically meaningful patient subgroups.

### 8.8. Future Research Priorities

Several research priorities emerge from the current evidence. First, multi-analyte biomarker panels that combine SCFAs, tryptophan metabolites, and markers of intestinal permeability should be prospectively validated as indicators of disease activity and treatment response. Second, clinical trials investigating targeted metabolite supplementation, including butyrate- and AhR-based interventions, are warranted. Third, harmonized international multi-omics cohorts are needed to improve reproducibility and facilitate cross-population comparisons. Finally, AI-assisted endotype classification systems integrating microbiome, metabolomic, and clinical data may represent a critical step toward precision microbiome medicine in AD.

## 9. Conclusions

Current evidence supports a biologically plausible role for the gut microbiome in modulating immune regulation, epithelial barrier function, and microbial metabolite signaling in AD. Human studies have identified associations between dysbiosis, altered SCFA and tryptophan-derived metabolite profiles, intestinal barrier dysfunction, and disease severity, while experimental models provide complementary mechanistic support. However, substantial heterogeneity across age groups, AD phenotypes, geographic populations, analytical platforms, and treatment exposures limits causal inference and immediate clinical translation. Microbiome-targeted interventions show variable and context-dependent effects, whereas FMT, WMT, postbiotics, and metabolite-directed approaches remain investigational. Future studies should prioritize longitudinal multicenter cohorts, standardized analytical methods, phenotype-aware patient stratification, clinically meaningful outcome thresholds, and prospective biomarker validation before microbiome-based precision strategies can be incorporated into routine AD care.

## Figures and Tables

**Table 1:** Representative gut-derived metabolites and intestinal barrier-associated markers investigated in relation to atopic dermatitis severity [36,68].

Biomarker or Metabolite	Biological Sample	Population and Age Group	Study Design	Sample Size	Direction of Association	Effect Estimate	Clinical Outcome	Principal Limitation	Clinical Readiness
Caproic acid	Plasma	Adults with active AD, age 18–50 years, and matched healthy controls	Cross-sectional case–control study	50 AD patients, 25 controls, correlations within the AD group	Lower levels are associated with greater disease severity	EASI: rho = −0.59, *p* < 0.001; Total SCORAD: rho = −0.42, *p* = 0.002; SCORAD extent: rho = −0.60, *p* = 0.001	EASI; total SCORAD; SCORAD extent	Cross-sectional design, small adult cohort, no causal inference, exploratory biomarker panel	Research only, not validated for routine clinical use
Isocaproic acid	Plasma	Adults with active AD, age 18–50 years, and matched healthy controls	Cross-sectional case–control study	50 AD patients, 25 controls, correlations within the AD group	Lower levels are associated with greater disease severity	EASI: rho = −0.31, *p* = 0.028; Total SCORAD: rho = −0.36, *p* = 0.010; SCORAD extent: rho = −0.36, *p* = 0.010; Extent during remission: rho = −0.50, *p* < 0.001	EASI; total SCORAD; SCORAD extent, extent during remission	Cross-sectional design, small adult cohort, no external validation	Research only, not validated for routine clinical use
SCFA factor cluster, butyric acid, valeric acid, and isocaproic acid	Plasma	Adults with active AD, age 18–50 years	Cross-sectional case–control study with factor analysis	50 AD patients, correlations within the AD group	Higher cluster values associated with lower severity	EASI: rho = −0.32, *p* = 0.021; EASI intensity: rho = −0.39, *p* = 0.005; Total SCORAD: rho = −0.28, *p* < 0.05; Extent during remission: rho = −0.42, *p* = 0.002	EASI; EASI intensity; total SCORAD; extent during remission	Factor-derived cluster, not a standalone validated biomarker; cross-sectional association	Research only, not validated for routine clinical use
Propionic acid and isovaleric acid factor cluster	Plasma	Adults with active AD, age 18–50 years	Cross-sectional case–control study with factor analysis	50 AD patients, correlations within AD group	Higher cluster values associated with lower residual disease extent	Extent during remission: rho = −0.29, *p* = 0.041	Extent of skin lesions during remission	Limited to one clinical outcome, factor-derived cluster, cross-sectional design	Research only, not validated for routine clinical use
Acetic acid	Infant plasma	Infants from the Swedish NICE birth cohort	Prospective birth cohort	148 infants	Higher plasma concentration at 4 months associated with lower odds of later atopic eczema	Adjusted OR per SD for atopic eczema at 12 months: 0.42, 95% CI 0.18–0.95	Atopic eczema at 12 months	Single cohort, early-life population only, outcome not directly comparable with adult AD severity scores	Research only, not validated for routine clinical use
Caproic acid	Infant plasma	Infants from the Swedish NICE birth cohort	Prospective birth cohort	148 infants	Higher plasma concentration at 4 months is associated with lower odds of subsequent sensitization	Adjusted OR per SD for sensitization at 12 months: 0.25, 95% CI 0.09–0.66	Sensitization at 12 months	Outcome was sensitization, not AD severity; early-life population only	Research only, not validated for routine clinical use
Indoxyl	Plasma	Adults with active AD, age 18–50 years, and matched healthy controls	Cross-sectional case–control study	50 AD patients, 25 controls, correlations within the AD group	Higher levels are associated with selected severity measures	EASI: rho = 0.31, *p* = 0.027; EASI extent: rho = 0.33, *p* = 0.018; SCORAD extent: rho = 0.37, *p* = 0.009; Total SCORAD: rho = 0.23, *p* = 0.11	EASI, EASI extent, SCORAD extent, total SCORAD	Association not significant for total SCORAD; cross-sectional exploratory finding	Research only, not validated for routine clinical use
Lipopolysaccharide-binding protein, LBP	Serum	Adults with active AD, age 18–50 years, and matched healthy controls	Cross-sectional case–control study	50 AD patients, 25 controls, correlations within the AD group	Higher levels are associated with greater disease severity	EASI: rho = 0.31, *p* = 0.027; Total SCORAD: rho = 0.32, *p* = 0.022; Objective SCORAD: rho = 0.36, *p* = 0.011; SCORAD extent: rho = 0.40, *p* = 0.004	EASI, total SCORAD, objective SCORAD, SCORAD extent	Non-specific marker of bacterial translocation/systemic inflammation; cross-sectional association	Research only, not validated for routine clinical use
Regenerating family member 3 alpha, Reg3A	Serum	Adults with active AD, age 18–50 years, and matched healthy controls	Cross-sectional case–control study	50 AD patients, 25 controls, correlations within the AD group	Higher levels are associated with greater disease severity	EASI: rho = 0.35, *p* = 0.014; Total SCORAD: rho = 0.31, *p* = 0.030; SCORAD extent: rho = 0.40, *p* = 0.004	EASI, total SCORAD, SCORAD extent	Pleiotropic epithelial and antimicrobial marker; not specific to AD or intestinal permeability	Research only, not validated for routine clinical use
Interleukin-10, IL-10	Serum	Adults with active AD, age 18–50 years, and matched healthy controls	Cross-sectional case–control study	50 AD patients, 25 controls, correlations within the AD group	Higher levels are associated with greater disease severity	EASI: rho = 0.35, *p* = 0.013; Total SCORAD: rho = 0.30, *p* = 0.034; SCORAD extent: rho = 0.42, *p* = 0.003	EASI, total SCORAD, SCORAD extent	Pleiotropic cytokine, not specific to gut barrier dysfunction; interpretation depends on cellular source and inflammatory context	Research only, not validated for routine clinical use
Interleukin-22, IL-22	Serum	Adults with active AD, age 18–50 years, and matched healthy controls	Cross-sectional case–control study	50 AD patients, 25 controls, correlations within the AD group	Higher levels are associated mainly with disease extent	EASI extent: rho = 0.37, *p* = 0.009; SCORAD extent: rho = 0.35, *p* = 0.013; Total SCORAD: rho = 0.26, *p* = 0.07	EASI extent, SCORAD extent, total SCORAD	Association not significant for total SCORAD; cytokine reflects broader epithelial and inflammatory signaling	Research only, not validated for routine clinical use

Representative gut-derived metabolites and intestinal barrier-associated markers investigated in relation to atopic dermatitis severity. The table summarizes study context and reported associations without applying a formal evidence-grading system. Clinical readiness refers to whether the marker has been validated for routine clinical use; none of the listed candidates should be considered an established standalone biomarker unless explicitly supported by prospective validation. Legend: AD, atopic dermatitis; EASI, Eczema Area and Severity Index; SCORAD, SCORing Atopic Dermatitis; LBP, lipopolysaccharide-binding protein.

## Data Availability

The data used to support the findings of this study are available from the corresponding author upon reasonable request.
